# SARS-CoV-2 Aerosol and Surface Detections in COVID-19 Testing Centers and Implications for Transmission Risk in Public Facing Workers

**DOI:** 10.3390/ijerph20020976

**Published:** 2023-01-05

**Authors:** Sarah J. Stein, Ashley R. Ravnholdt, Vicki L. Herrera, Danielle N. Rivera, Paul T. Williams, Joshua L. Santarpia

**Affiliations:** 1 Department of Pathology and Microiology, University of Nebraska Medical Center, Omaha, NE 68106, USA; 2 Global Center for Health Security, University of Nebraska Medical Center, Omaha, NE 68106, USA; 3 National Strategic Research Institute, Omaha, NE 68106, USA

**Keywords:** SARS-CoV-2, environmental contamination, infection risk

## Abstract

The severe acute respiratory syndrome coronavirus 2 (SARS-CoV-2) and resulting COVID-19 (coronavirus disease 2019) pandemic have required mass diagnostic testing, often taking place in testing sites within hospitals, clinics, or at satellite locations. To establish the potential of SARS-CoV-2 aerosol transmission and to identify junctures during testing that result in increased viral exposure, aerosol and surface samples were examined for the presence of SARS-CoV-2 RNA from locations within Nebraska Medicine COVID-19 testing and vaccine clinics. Aerosols containing SARS-CoV-2 RNA detected within clinics suggest viral shedding from infected individuals. SARS-CoV-2 RNA detection in aerosol samples was shown to correlate with clinic operation and patient infection, as well as with community infection findings. Additionally, SARS-CoV-2 RNA was detected in surface samples collected from clinics. The presence of SARS-CoV-2 RNA in aerosols in these clinics supports the continued use of respiratory protection and sanitization practices for healthcare workers, and other workers with public facing occupations.

## 1. Introduction

COVID-19 has resulted in significant human morbidity and mortality from 2019 to present. The virus rapidly spread worldwide and was officially declared a global pandemic by the World Health Organization (WHO) on 11 March 2020 [1]. As of November 2022, COVID-19 has infected over 638 million people and has led to over 6.6 million deaths [2]. Nosocomial infections in hospitals and treatment settings have been an ongoing concern due to the high degree of exposure of healthcare workers [3]. Highly trafficked public locations within communities such as public transportation, grocery stores, post-offices, restaurants, and Transportation Security Administration (TSA) security checkpoints are also likely sources of infection.

Respiratory viruses, including SARS-CoV-2, can infect their hosts through aerosols [4,5]. Additionally, while less supported than respiratory transmission, there has been some concern for the possibility of contact/fomite transmission of SARS-CoV-2 [6]. Aerosol transmission has been reported as the dominant mode of transmission for SARS-CoV-2 [4,7]. Infected individuals release virion-containing aerosols as they cough, sneeze, exhale, or speak, and SARS-CoV-2 was found to remain viable and infectious in aerosols smaller than 5 μm for many hours [8]. Multiple studies have found positive SARS-CoV-2 RNA air samples from locations such as standard hospital rooms and negative pressure equipped medical rooms, supporting the need for airborne precautionary measures [9,10]. Additionally, respiratory personal protective equipment (PPE) use was widely extended to the general public as a precautionary measure for reducing transmission and infection, with varying levels of efficacy depending on filter material [11]. Retrospective studies for SARS-CoV-2 have indicated that airborne transmission would explain the number of secondary infections that have occurred from viral clustering patterns [12,13,14,15]. SARS-CoV-2 transmission due to fomite contamination has also been hypothesized as a plausible transmission route, as the virus can survive for multiple days on a range of surface materials [8,16].

Considering the highly contagious nature of SARS-CoV-2, safety concerns for individuals who work with the public, such as health care workers, have increased [17]. Prior studies have proposed that healthcare workers and patients have become infected with SARS-CoV-2 from visiting hospitals [18]. Additionally, transmission risks associated with highly trafficked locations are public health concerns. COVID-19 testing and observation sites within hospitals, clinics, or at satellite locations are ideal for investigating this broader issue. All healthcare workers interacting with patients at COVID-19 testing sites from the time of check-in to the time of collecting the patient’s sample are at increased risk of SARS-CoV-2 exposure, as these clinics amass a population of people possibly infected with SARS-CoV-2, which necessitates significant protective measures and PPE use. Testing procedures which take place at these clinics usually entail nasal or oral swabs and/or saliva tests. The handling of infectious biological samples is potentially hazardous in the absence of PPE and sanitization measures, and the potential creation of bioaerosols and infectious fomites may translate to an increased risk of exposure for healthcare workers and patients alike.

Additionally, the continual emergence of new SARS-CoV-2 variants requires continued surveillance and attention. For much of this study, the dominant strain in the U.S. was the Delta variant, and during this study was classified as a variant of concern by the World Health Organization (WHO) [19,20]. The Delta variant is defined by amino acid changes in the spike protein which resulted in increased transmissibility and infectivity due to high viral loads in infected individuals and shortened generation time [19,21,22,23]. Patients who contract the Delta variant of SARS-CoV-2 have a higher risk of hospitalization, requirement of intensive care, and mortality when compared to previous variants [19]. These observations, coupled with decreased preventative measures, could account for surges in case counts. The more recent Omicron variants are currently the dominant strain in both the U.S. and the world at the time of this study, and they are also classified as variants of concern by the WHO [20,24]. While thought to result in less severe illness, the Omicron variants have displayed an increased transmission rate [25].

This study analyzes the possible exposures of SARS-CoV-2 that healthcare workers and patients face at COVID-19 testing and vaccination clinics located at a hospital and a satellite location. Locations like this serve high traffic, transient populations which may include SARS-CoV-2 infected individuals in passing. Additionally, these locations were utilized to identify junctures during COVID-19 testing, which could potentially create increased vulnerability. Aerosol and surface samples were collected to examine the presence of SARS-CoV-2 in the air and on fomites, which were used to draw relevant conclusions about the subsequent possibilities of exposure. While these findings are from testing and treatment locations where PPE is used to minimize potential exposures, they may also offer insights into the potential for exposures in highly trafficked, community-serving locations.

## 2. Materials and Methods

**Figure 1:**
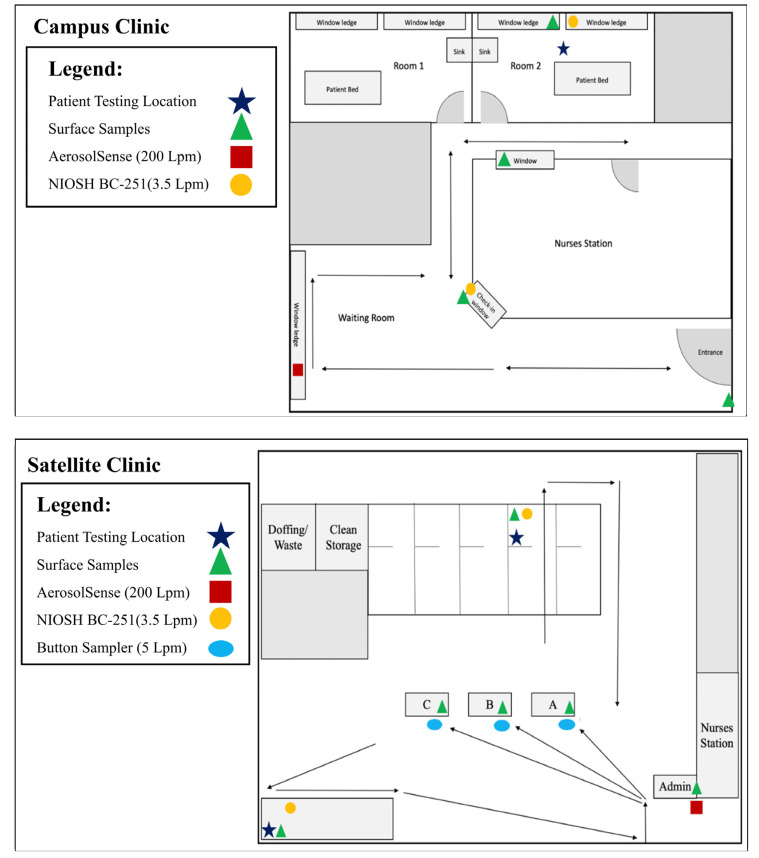
Schematics of sampling sites. Top panel depicts the campus clinic and bottom panel depicts the satellite clinic. Arrows indicate patient flow during entrance, check in, COVID-19 test collection, and exit. Legend indicates sample type collected at check-in desks and test collection locations within each clinic. In the satellite clinic, the only overlap of patient flow occurs near the entrance/exit and immediately inside the building. In the campus clinic, there is a high level of overlap in patient flow, as hallways are used for both entrance and exit.

Samples were collected in collaboration with Nebraska Medicine COVID-19 testing and vaccination clinics. Testing was conducted at two sites: a testing clinic on campus at the University of Nebraska Medical Center (hereafter referred to as the campus clinic; Figure 1 (top)), and a Nebraska Medicine satellite testing and vaccination clinic within the community (hereafter referred to as satellite clinic; Figure 1 (bottom)). The campus clinic was located within a hospital clinic space (~465 m^2^), complete with a waiting room and exam rooms, whereas the satellite clinic was located in a vacant supermarket outfitted for clinic use (~7900 m^2^). Patients entering testing clinics were required to don face masks while inside the clinic, with the exception of the examination room while being tested. Aerosol and surface samples were collected from highly trafficked areas within each location, such as check-in areas, waiting areas, and specimen collection areas (Figure 1). Sampling in the satellite clinic occurred between 29 June 2021–27 July 2021 and in the campus clinic from 7 September 2021–29 October 2021. The Delta variant was dominant (greater than 50% of sequenced cases) in Nebraska throughout this period [26].

Air sampling was done using three types of aerosol samplers: an AerosolSense 2900 sampler (ThermoFisher Scientific, Waltham, MA, USA) operating at 200 Lpm, National Institute of Occupational Safety and Health (NIOSH) BC-251 2-stage cyclone samplers with vacuum supplied by an Airchek pump (Model 224-44XR, SKC, Eighty Four, PA, USA) at 3 Lpm, and Button Samplers (SKC, Eighty Four, PA, USA) with vacuum supplied by an Airchek XR5000 pump (SKC, Eighty Four, PA, USA) at 5 Lpm [27,28,29,30]. Sampling time ranged from between 3.5–120 h, depending on clinic location and sampler type.

NIOSH BC-251 are particle size selective samplers that segregate particles into 3 size ranges: aerodynamic diameter (d_a_) > 4.4 μm, d_a_ = 1.1–1.4 μm, and da < 1.1 μm. The first stage collects into a 15 mL tube (d_a_ > 4.4 μm), followed by a 1.5 mL tube (d_a_ = 1.1–1.4 μm), and finally a 37 mm Gelatin Filter (Sartorius Stedim Biotech GmbH, Goettingen, Germany; d_a_ < 1.1 μm) [27]. NIOSH BC-251 samplers were placed at the check-in station and/or examination room, depending on location, within approximately two feet of the patients in both locations. Exclusively at the satellite clinic, SKC Button Samplers were placed at the individual check-in desks and collected into 25 mm Gelatin Filter (Sartorius Stedim Biotech GmbH, Goettingen, Germany). Surface samples were collected from hand sanitizer pumps, door handles, the check in station, and the examination room using 9 in2 sterile gauze in 3 mL sterile 1 × phosphate-buffered saline (PBS) over a 100 cm^2^ surface.

All aerosol samplers (SKC Button sampler, NIOSH BC-251, and AerosolSense) were run within the satellite clinic for 3.5 h simultaneously. SKC Button sampler and NIOSH BC-251 were run within the campus clinic for 5 h simultaneously. For the duration of sampling periods within the campus clinic, AerosolSense ran once for 24 h (6 October–7 October), twice for 48 h (20 September–22 September and 27 September–29 September), once for 96 h (25 October–29 October), and once for 120 h (20 October–25 October).

NIOSH BC-251 samples were recovered 1–2 h after collection by adding 1 mL sterile PBS each to the 15 mL and 1.5 mL tubes and rinsing the walls thoroughly via pipette mixing and vortexing for 10 s. Last stage 37 mm gelatin filters were dissolved in 5 mL sterile 1× PBS at 37 °C in a 50 mL conical tube and vortexed for 10 s to establish homogeneity. SKC Button samplers were recovered by dissolving 25 mm gelatin filters in warmed 5 mL sterile 1× PBS in a 50 mL conical tube and vortexing 10 s to establish homogeneity. AerosolSense collection substrates were removed from their cartridges using sterile tweezers and recovered in 1 mL sterile PBS using a HSW Soft-Ject disposable syringe to squeeze recovery fluid from the sponge substrates. Surface samples were rehydrated in an additional 10 mL sterile 1× PBS. All samples were shaken vigorously and vortexed for 10 s for maximum virus recovery.

RNA extraction was performed immediately after recovery using a Qiagen EZ1 Advanced XL and Qiagen Virus Mini Kit v2.0 (QIAGEN GMbH, Hilden, Germany). An initial sample (400 µL) was used for extraction for sample elution in 60 µL of Qiagen Buffer AVE. To detect SARS-CoV-2 genomic RNA, a reverse-transcription quantitative polymerase chain reaction (RT-qPCR) assay targeting the SARS-CoV-2 E gene was used [31]. The target primer/probe and target sequences are listed below:

E gene target primers and probe:

Probe: 5′/56-FAM/ACACTAAGCC/ZEN/ATCCTTACTGCGCTTCG/3AIBkFG/-3′
Primer 1: 5′-ATATTGCAGCAGTACGCACACA-3′
Primer 2: 5′-ACAGGTACGTTAATAGTTAATAGCGT-3′

ssDNA E Target Sequence:

5′-TTCGGAAGAGACAGGTACGTTAATAGTTAATAGCGTACTTCTTTTTCTTGCTTTCGTGGTATTCTTGCTAGTTACACTAGCCATCCTTACTGCGCTTCGATTGTGTGCGTACTGCTGCAATATTGTTAACGTG-3′

Reverse Transcriptase-Polymerase Chain Reaction (RT-PCR) was performed using Invitrogen Superscript III Platinum One-Step Quantitative RT-PCR System. Each PCR run included a viral RNA positive control and a negative, no template, control of nuclease free water. Reactions were set up and run with initial conditions of 10 min at 55 °C and 4 min at 94 °C then 45 cycles of 94 °C for 15 s and 58 °C for 30 s on a QuantStudio 3 thermocycler (Applied Biosytems, Inc, Waltham, MA, USA.) utilizing the following reagents:

5.6 µL nuclease free water;
12.5 µL Invitrogen 2X Master Mix;
0.4 µL MgSO4;
1.0 µL Primer/Probe Mix (IDT) × (Primers 10 µM, Probe 5 µM);
0.5 µL SuperScript III Platinum Taq;
5.0 µL extracted sample RNA, nuclease free water or positive control;
25.0 µL Total.

As an additional control, blank samples of hydrated gauze and gelatin filters, both carried during sampling or kept in the laboratory, were analyzed. No amplification of blank samples was observed.

A 6-log standard curve run in triplicate using synthetic DNA was used to quantify virus from each sample using cycle time (Ct) obtained from RT-qPCR. The data were fit with the following exponential function:

(1) $$\frac{\mathrm{copies}}{\mathrm{mL}}=9.0\times 10^{12}e^{-0.554*\mathrm{Ct}}$$

Samples were run in triplicate. Average sample Ct was used in the equation above to calculate average copies/mL. Undetected samples were evaluated at zero before calculating the average concentration. The maximum Ct value that was accepted in this study was 39, which is consistent with prior work [10]. The minimum detectable concentration for each sample was calculated to be 1241 copies/mL, assuming that only one of three replicates amplified at a Ct of 39.

Patient medical records were not accessed for the purposes of this study. Aggregate statistics were obtained from clinics, including number of patients tested per day and number of positive tests per day. A data summary from the campus clinic including number of tests taken per hour and number of positive tests per hour was made available by Nebraska Medicine for the purposes of this study. Data available from the satellite clinic were limited to number of tests taken and number of patients reporting symptoms upon check-in.

Because the medical campus serves not only the city of Omaha (located in Douglas County), but the surrounding metropolitan area, data were gathered for Douglas, Sarpy, and Washington County as well as a metropolitan area including Council Bluffs, IA. The COVID ActNow U.S. COVID Risk and Vaccine Tracker [32] was utilized to gather community COVID-19 positivity and test positivity rates for these communities.

Where applicable, linear regression analysis for ‘copies/L of air’ against variables ‘total patients tested’, ‘positive patients’, and ‘percentage of positive patients’ was completed to identify potential relationships between measured concentrations of SARS-CoV-2 in aerosol and the population visiting the clinic. A Pearson’s R > 0.6 were considered to indicate strong correlation, 0.4 < R < 0.6 considered moderate, 0.1 < R < 0.4 considered weak, and < 0.1 considered to indicate no correlation. Statistical analysis was done using Microsoft Excel™ Version 16.60 (22041000).

## 3. Results

Aerosol and surface sampling was conducted in both satellite and campus clinics to detect SARS-CoV-2 RNA positivity. In addition, tests administered per day and patient positivity statistics were obtained from clinics and positivity and test positivity rates were obtained from the surrounding community. Linear regressions between environmental observations and clinic test data, as well as comparisons between clinic positivity data and surrounding community data were performed.

### 3.1. Satellite Clinic

Over the course of ten sampling dates, surface testing was performed on the hand sanitizer, check-in station, and testing room. Air testing was conducted at the check-in tables and testing/collection room, with AerosolSense air testing conducted at the front entrance. During sampling, the satellite clinic tested between 16–23 total patients a day which included 6–18 symptomatic patients.

Three surface samples were positive for SARS-CoV-2 RNA: two at check-in stations and one in the testing room itself (copies/cm^2^ = 194.94, 388.33, and 165.27, respectively) (Table 1). One positive air sample was collected via Button Sampler at the check-in station (copies/L of air = 0.22) (Table 1). No positive samples were collected by the AerosolSense (Table 1).

### 3.2. Campus Clinic

During the seven weeks of sampling, surface testing was done at the check-in desk, hand sanitizer, entrance door handle, and exam room. Air testing was conducted at the check-in desk and exam room using NIOSH BC-251. The AerosolSense collected air from the waiting area. During sampling the campus clinic administered between 31–111 total COVID-19 tests per day, including between 1–13 positive tests. As one test is equivalent to one patient, AerosolSense sampling periods occurred during the testing of between 145–422 total patients, including an estimated 11–32 positive patients.

Two positive surface samples were collected: once for the hand sanitizer and once at the check-in desk (copies/cm^2^ = 269.11, copies/cm^2^ = 303.99, respectively) (Table 2). One positive air sample was collected via NIOSH BC-251 sampler at the check-in desk (copies/L of air = 17.14) (Table 2). AerosolSense testing yielded positive results during every sampling period (Table 2).

There was a strong anti-correlation between copies/L of air vs. total patients tested in the campus clinic (R = −0.63, R^2^ = 0.39) and copies/L of air vs. positive patients tested at the campus clinic (R = −0.65, R^2^ = 0.43). There was a weak anti-correlation between copies/L of air vs. the percentage of positive patients tested at the campus clinic (R = −0.17, R^2^ = 0.03).

Positivity in the campus clinic test data was compared to test positivity rates and community positivity rates to provide context to the collected environmental data when extending the results to a broader range of frontline workers. In general, test positivity rates from the surrounding community were between 1.5 and 3.5 times higher than from the campus clinic (Table 2). Community positivity rates were much lower than test positivity rates in the clinic. Over all the weeks examined the percentage of COVID-19 positive individuals passing through the clinic was 39.6 ± 12.4 times higher than the percentage of COVID-19 positive individuals in the community at large (Table 2).

## 4. Conclusions

The distinction between the physical spaces occupied by each clinic is important when interpreting aerosol sampler results. The satellite clinic had high ceilings and spatial openness characteristic of a supermarket. In contrast, the campus clinic was more office-like, with lower ceilings and smaller rooms. Greater spatial volume of the satellite clinic may account for fewer observations of aerosol contamination. In addition to the dynamics of the sampling space, the satellite clinic also tested fewer patients daily in comparison to the campus clinic, which likely affected aerosol testing results. Various surface samples were positive for SARS-CoV-2 RNA at both the satellite and campus clinics.

Within the campus clinic, strong anti-correlations existed between copies/L of air, measured by the AerosolSense vs. total patients tested and vs. positive patients tested. This suggest that weeks with both fewer positive patients and fewer patients overall resulted in more SARS-CoV-2 contamination in the air, which is counterintuitive. Since mitigation techniques for aerosol, such as portable HEPA filtration, were not in use during this study, changes in cleaning and mitigation strategies during low traffic periods do not likely explain these results. However, several other considerations must be made to correctly interpret the data. Since the number of positive patients is strongly correlated with the total number of patients (R = 0.97), but the percentage of positive patients is only weakly correlated (R = 0.28) with total number of patients, the number of positive patients does not increase proportionally with the total number of patients tested in a given week. Since this clinic is used both for testing suspected COVID-19 cases and for screening patients prior to surgical procedures, the clinic traffic itself may driven largely by fluctuations in the hospital surgical schedule. This is further indicated in lower test positivity rates in this clinic, compared to the surrounding community test positivity rates.

The weak anti-correlation between copies/L of air vs. percentage of positive patients, suggests that AerosolSense positivity was more dependent on select individual exposure events, rather than an accumulation of SARS-CoV-2 aerosol from all positive patients (Table 2). This could potentially indicate that the rate at which infected individuals shed virus may play a larger role than the number of individuals passing through the space. This minimizes concern for patients in the waiting room or exposed to clinic air for brief durations, which is indicated by the prevalence of positive detection from long-term sampling. Furthermore, these routine observations of viral aerosol could not be directly implicated in causing disease. The mandatory use of respiratory protection by both healthcare workers and patients and routine cleaning procedures likely also mitigated risk.

Viral RNA collected in the NIOSH BC-251 filter at the campus clinic indicates that particles with aerodynamic diameter of <1.1 μm containing SARS-CoV-2 RNA were shed from patients checking in for testing (Table 2). This further indicates that aerosols of a small size can carry SARS-CoV-2. This is consistent with previous observation and isolation of SARS-CoV-2 from small respirable particles [33,34], and further supports the role of aerosols in transmission. The single positive NIOSH BC-251 filter, along with the fact that this observation did not coincide with elevated patient positivity rates, indicates that this was likely due to isolated viral shedding instance. Similarly, the isolated observation of SARS-CoV-2 on the SKC Button sampler filter at the satellite clinic indicated an occurrence of viral shedding.

Detection by both long- and short-term aerosol sampling highlight the potential risks involved for healthcare personnel and first responders when interacting with infected individuals, supporting the use of high-quality respiratory PPE (such as N95 respirators) for employees during interaction periods. When extending these results to risks for frontline workers interacting with the community, a variety of considerations must be made. First, it is clear that community testing sites that focus on testing suspected COVID-19 cases, and whose percentage of positive tests is higher than those in the clinic encounter more COVID-19 positive individuals, and are therefore more likely to encounter an individual shedding high amounts of virus. These clinic settings also allow us to draw conclusions about the type of exposure profile which may apply to other public facing jobs. The total recorded number of individuals processed through the campus clinic varied between 36 and 97 per day, with COVID-19 positivity rates between 5.9% and 7.8% (during periods where the AerosolSense was operating). This is 39.6 ± 12.4 times higher than the community positivity rates. This indicates that a location that encounters between approximately 1000 and 5000 individuals in the public per day, would have similar likelihood of observation of SARS-CoV-2 aerosol, and its associated risk. This applies to locations such as public transportation, TSA security checkpoints, and community serving locations such as grocery stores, department stores and similar large retail locations. Therefore, respiratory protection should be recommended for workers in those locations, during periods of enhanced community transmission.

The contamination of frequently touched objects such as hand sanitizers and desktop surfaces demonstrate the concern of SARS-CoV-2 transmission via fomites, as SARS-CoV-2 has shown to be viable on surfaces [8,16,35]. Contamination of these types of surfaces is expected in a COVID-19 testing clinic, and supports periodic surface decontamination practices to reduce risk of potential exposure. It is important to note, that while surfaces are not considered a dominant mode of transmission for COVID-19 [36], there is still potential risk, particularly in healthcare and testing environments, where higher levels and increased frequency of surface contamination can be expected.

Studies suggest that viral shedding through respiratory emissions varies between individuals [37,38]. Combined with the observations of this study, those findings suggest long-term sampling strategies may be more effective at estimating risk in indoor spaces. The short-duration measurements by the BC-251, Button Sampler, and AerosolSense in the satellite clinic, observed only intermittent positive detections, despite the consistent traffic of SARS-CoV-2 positive individuals. However, multi-day sampling by the AerosolSense in the campus clinic identified SARS-CoV-2 in aerosols on multiple occasions. Given the variability of viral shedding, particularly in respiratory emissions, long-duration sampling will be more effective at identifying areas where risk of exposure to SARS-CoV-2 aerosol is more likely.

This study demonstrates contamination of air within testing clinics with viral RNA, as well as occasional contamination of frequently touched objects. Use of respiratory PPE for both potentially COVID-19 positive individuals and those that may routinely interact with them is supported by the aerosol sampling results. These results can be extended to other public facing workers, in a variety of industries, by examining the community positivity rate and the number of individuals encountered by that worker population.

## Figures and Tables

**Table 1:** Summary of RT-qPCR Ct values collected from the Nebraska Medicine satellite clinic. ND indicates non-detected samples. If all three technical triplicates were ND a summary result is provided. If mixed or quantitative detection was observed, the individual results were reported.

Date	Surface						Air										Symptomatic Patients	Total Patients
	Hand Sanitizer		Check-In		Testing Room		Check-In Station		Testing/Collection Room						AerosolSense			
									<1 µm		1–4 µm		>4.1 µm					
	Ct	Copies/cm^2^	Ct	Copies/cm^2^	Ct	Copies/cm^2^	Ct	Copies/L of Air	Ct	Copies/L of Air	Ct	Copies/L of Air	Ct	Copies/L of Air	Ct	Copies/L of Air		
29 June 2021	ND		37.78 ND ND	194.94	ND		35.84 ND ND	0.22	ND		ND		ND		ND		6	52
30 June 2021	ND		ND		ND		ND		ND		ND		ND		ND		8	50
1 July 2021	ND		ND		ND		ND		ND		ND		ND		ND		8	16
6 July 2021	ND		ND		ND		ND		ND		ND		ND		ND		None	38
7 July 2021	ND		ND		ND		ND		ND		ND		ND		ND		6	53
8 July 2021	ND		ND		ND		ND		ND		ND		ND		ND		18	34
13 July 2021	ND		ND		38.08 ND ND	165.27	ND		ND		ND		ND		ND		13	42
14 July 2021	ND		37.71 37.87 ND	388.33	ND		ND		ND		ND		ND		ND		5	43
15 July 2021	ND		ND		ND		ND		ND		ND		ND		ND		10	23
27 July 2021	ND		ND		ND		ND		ND		ND		ND		ND		17	35

**Table 2:** Summary of RT-qPCR Ct values collected from the Nebraska Medicine campus clinic, test positivity rates from the clinic and the surrounding community, and community positivity rates per 100,000 individuals. ND indicates non-detected samples; NC indicates that the value was not calculated; N/A indicates the valuable in not applicable.

Date	Surface								Air														Clinic Positive Tests			Percentage of Positive Tests			Percent Community Positivity			
	Hand Sanitizer		Check-In		Door Handle		Exam Room		Check-In						Exam Room						AerosolSense		Positive COVID-19 Tests	Total COVID-19 Tests	Percent	Douglas County	Sarpy County	Washington County	Douglas County	Sarpy County	Washington County	Omaha Metro
									<1 µm		1–4 µm		>4.1 µm		<1 µm		1–4 µm		>4.1 µm													
	Ct	Copies/cm^2^	Ct	Copies/cm^2^	Ct	Copies/cm^2^	Ct	Copies/cm^2^	Ct	Copies/L of Air	Ct	Copies/L of Air	Ct	Copies/L of Air	Ct	Copies/L of Air	Ct	Copies/L of Air	Ct	Copies/L of Air	Ct	Copies/L of Air										
7 September 2021	ND		ND		ND		ND		ND		ND		ND		ND		ND		ND		N/A		5	46	10.87%	NC	NC	NC	NC	NC	NC	NC
20 September 2021	37.20 ND ND	269.11	ND		ND		ND		ND		ND		ND		ND		ND		ND		N/A		3	36	8.33%	NC	NC	NC	NC	NC	NC	NC
20–22 September 2021	N/A		N/A		N/A		N/A		N/A		N/A		N/A		N/A		N/A		N/A		35.08 35.27 36.11	0.047	11	145	7.59%	11.80%	13.30%	19.10%	0.19%	0.28%	0.10%	0.23%
27 September 2021	ND		ND		ND		ND		38.33 ND ND	17.14	ND		ND		ND		ND		ND		N/A		4	91	4.40%	NC	NC	NC	NC	NC	NC	NC
28 September 2021	ND		ND		ND		ND		ND		ND		ND		ND		ND		ND		N/A		4	82	4.88%	NC	NC	NC	NC	NC	NC	NC
29 September 2021	ND		ND		ND		ND		ND		ND		ND		ND		ND		ND		N/A		7	85	8.24%	NC	NC	NC	NC	NC	NC	NC
27–29 September 2021	N/A		N/A		N/A		N/A		N/A		N/A		N/A		N/A		N/A		N/A		35.39 35.92 37.49	0.033	18	307	5.86%	11.70%	12.70%	20.80%	0.17%	0.21%	0.22%	0.19%
6 October 2021	ND		ND		ND		ND		ND		ND		ND		ND		ND		ND		N/A		4	94	4.26%	NC	NC	NC	NC	NC	NC	NC
2021/10/7	ND		ND		ND		ND		ND		ND		ND		ND		ND		ND		N/A		8	97	8.25%	NC	NC	NC	NC	NC	NC	NC
6–7 October 2021	N/A		N/A		N/A		N/A		N/A		N/A		N/A		N/A		N/A		N/A		37.62 ND ND	0.009	12	191	6.28%	11.90%	12.00%	11.10%	0.17%	0.21%	0.23%	0.19%
21 October 2021	ND		ND		ND		ND		ND		ND		ND		ND		ND		ND		N/A		6	45	13.33%	NC	NC	NC	NC	NC	NC	NC
22 October 2021	ND		36.98 ND ND	304	ND		ND		ND		ND		ND		ND		ND		ND		N/A		5	57	8.77%	NC	NC	NC	NC	NC	NC	NC
20–25 October 2021	N/A		N/A		N/A		N/A		N/A		N/A		N/A		N/A		N/A		N/A		37.72 ND ND	0.002	30	387	7.75%	11.40%	11.40%	12.80%	0.15%	0.19%	0.18%	0.15%
25–29 October 2021	N/A		N/A		N/A		N/A		N/A		N/A		N/A		N/A		N/A		N/A		35.67 37.39 ND	0.009	32	422	7.58%	13.20%	12.40%	13.70%	0.16%	0.15%	0.17%	0.20%

## Data Availability

All data in this study are either derived from the cited, publicly available sources or presented in the manuscript.
